# Efficacy of Pitfall Trapping, Winkler and Berlese Extraction Methods for Measuring Ground-Dwelling Arthropods in Moist-Deciduous Forests in the Western Ghats

**DOI:** 10.1673/031.010.9801

**Published:** 2010-07-08

**Authors:** Thomas K. Sabu, Raj T. Shiju

**Affiliations:** Litter Entomology Research Unit, Post Graduate & Research Department of Zoology, St. Joseph's College, Devagiri, Calicut, Kerala, India 673008

**Keywords:** forest floor arthropods, soil-litter arthropods, quantitative and qualitative sampling, Acariformes, Araneae, Blattaria, Chalcidae, Chilopoda, Coleoptera, Dermaptera, Diplopoda, Diptera, Formicidae, Hemiptera, Homoptera, Isopoda, Isoptera, Larva, Lepidoptera, Mantoidea, Orthoptera, Hymenoptera, Parasitiformes, Phasmida, Pseudoscorpionida, Psocoptera, Thysanoptera

## Abstract

The present study provides data to decide on the most appropriate method for sampling of ground-dwelling arthropods measured in a moist-deciduous forest in the Western Ghats in South India. The abundance of ground-dwelling arthropods was compared among large numbers of samples obtained using pitfall trapping, Berlese and Winkler extraction methods. Highest abundance and frequency of most of the represented taxa indicated pitfall trapping as the ideal method for sampling of ground-dwelling arthropods. However, with possible bias towards surface-active taxa, pitfall-trapping data is inappropriate for quantitative studies, and Berlese extraction is the better alternative. Berlese extraction is the better method for quantitative measurements than the other two methods, whereas pitfall trapping would be appropriate for qualitative measurements. A comparison of the Berlese and Winkler extraction data shows that in a quantitative multigroup approach, Winkler extraction was inferior to Berlese extraction because the total number of arthropods caught was the lowest; and many of the taxa that were caught from an identical sample via Berlese extraction method were not caught. Significantly a greater frequency and higher abundance of arthropods belonging to Orthoptera, Blattaria, and Diptera occurred in pitfall-trapped samples and Psocoptera and Acariformes in Berlese-extracted samples than that were obtained in the other two methods, indicating that both methods are useful, one complementing the other, eliminating a chance for possible under-representation of taxa in quantitative studies.

## Introduction

Inclusion of ground-dwelling arthropods in biodiversity inventories and environmental assessment surveys has increased in the recent past (Oliver and Beattie 1996). Since most ground-dwelling arthropods are minute and numerous, and usually not easy to detect by an unaided eye, assessment of populations of these organisms is hard and labor-intensive. Specific methods that are effective in extracting a high proportion of these taxa are usually necessary. Three methods employed widely to survey ground- dwelling arthropods are pitfall trapping (Holland and Reynolds 2005; King and Porter 2005; Ward et al. 2001; Standen 2000; Brennan et al. 1999; Holland and Smith 1999; Mommertz et al.1996; Mesibov et al. 1995; Olson 1991; Adis 1979), Berlese extraction (Anu et al. 2009; Palacios-Vargas et al. 2007; Anu and Thomas 2006; King and Porter 2005; Richardson et al. 2005; Edwards 1991; Frith and Frith 1990), and Winkler extraction (Anto and Thomas 2007; Philpott et al. 2007; Robertson 2007; Vineesh et al. 2007; Krell et al. 2005; Leponce et al. 2004; Fisher and Robertson 2002; Longino et al. 2002; Chung et al. 2000; Fisher 1998; Beishaw and Bolton 1994).

Pitfall trapping, the simplest and cheapest method among the three, is efficient in capturing those arthropod taxa that are nocturnally active on the surface, but is inefficient in capturing either the bottom dwellers or those that disseminate by flying (Hansen and New 2005; Leather and Watt 2005; Woodcock 2005; Work et al. 2002; Ward et al. 2001; Standen 2000; Mommertz et al. 1996; Mesibov et al. 1995; Spence and Niemela 1994; Topping and Sunderland 1992; Adis 1979; Geenslade 1964). Pitfall trapping is most effective in open habitats, such as grasslands and scrub vegetation because the capture values can be affected by vegetation complexity (Melbourne 1999; Majer 1997; Greenslade 1964). Berlese extraction necessitates the use of expensive and unwieldy apparatuses and electricity, which may not be available in remote study sites (Krell et al. 2005; Lasebikan et al. 1978); moreover, in the Berlese extraction method, separation of soil particles and debris that drop into the collection solution along with fauna makes sampling more time-consuming and labour intensive than pitfall trapping (Robertson 2007; Edwards 1991). Winkler extraction is suitable for the extraction of litter-inhabiting, rapidly mobile Formicidae (Delabie et al. 2007; Underwood and Fisher 2006; Longino et al. 2002; Parr and Chown 2001; Bestelmeyer et al. 2000; Delabie et al. 2000; Olson 1991; Nadkarni and Longino 1990) and for the extraction of forest-litter inhabiting Coleoptera (Didham et al. 1998). However, Winkler extraction is less suitable for the extraction of all ground-dwelling arthropod taxa because chances for escape of the larger and more agile taxa are high; moreover, chances for the death of small taxa, with a narrow ecological tolerance, before they drop into the collection jars is also equally high (Didham et al. 1998; Besuchet et al. 1987). Winkler extraction is a relatively slow process in moist and humid environments, and when taxa of Formicidae are abundant in the sample, they may consume other fauna during extraction (Schillhammer 2001; Wheeler and McHugh 1987). Even for the litter-inhabiting Formicidae, for which the Winkler extraction method has proved suitable, it can extract a greater variety of taxa in warm-weather regions than it can in cold-weather regions (Leponce et al. 2004). Therefore, the relative efficiency of Winkler extraction in capturing ground-dwelling arthropods compared with the other sampling methods needs establishment.

In summary, the effectiveness of these three widely used methods to extract arthropods from soil substrates is being debated. Only a few replicated field studies have attempted to evaluate critically and compare quantitatively the extraction efficiency of the three sampling methods. Most of the earlier efforts evaluated the sampling efficiency of either the Berlese or the Winkler extraction method against pitfall trapping by sampling of a few specific arthropod taxa (e.g., pitfall trapping and Berlese extraction for Carabidae, Spence and Niemela 1994; Coleoptera, Formicidae and Araneae, Oliver and Beatie 1996; litter-inhabiting Formicidae, King and Porter 2005; Diplopoda, Snyder et al. 2006; Pitfall trapping and Winkler extraction for litter Formicidae, Fisher and Robertson 2002, Parr and Chown 2001, Delabie et al. 2000, Fisher 1999, Olson 1991.)

Hence, a considered opinion on the ideal sampling method for the extraction of the whole suite of ground-dwelling arthropods among the three remains to be established. Nonetheless, because of the easy manipulability in terms of time and cost effectiveness, pitfall trapping and Winkler extraction methods are being preferred over the Berlese extraction method in ecological surveys of soil arthropods (Krell et al. 2005; Chung et al. 2000; Didham et al. 1998; Belshaw and Bolton 1994; Spence and Niemela 1994; Hammond 1990; Ward 1987). These conclusions have been arrived at without evaluating the extraction efficiencies of the three methods although it is essential that any chosen method should address minimizing problems associated with complex statistical analysis, which could be compounded further by low numbers of taxa (Prasifka et al. 2007; Parr and Chown 2001).

In the present paper, the trapping efficiencies of the three widely used ground-dwelling arthropod trapping methods (pitfall, Berlese and Winkler extraction methods) were compared and evaluated using a field trial done in the moist deciduous forests in the Western Ghats in South India. The objective was to determine whether the three methods are equally effective in the separation and to determine which method achieves the best overall population numbers of as many taxa as possible.

## Materials and Methods

### Study area

The study was carried out in the moist deciduous forests of Sholayar (220 MASL, 20.55 km2) (10° 17′–10° 19′ N; 76° 39′–76° 44′ E), situated close to the Athirapally—Vazhachal waterfalls, 60 km south of the town of Trichur (Trichur District, Kerala State, India) located in the South-Western Ghats of moist deciduous ecoregion (Wikramanayake et al. 2002). Annual temperature 24–32° C; 40–80% RH; average rainfall 3,000–3,250 mm/year, which occurs mostly in June–November; June, July, and August receive the most rain.

### Sampling

Sampling was done in the first week of February 2006. Although a thorough population assessment of any group of invertebrates necessitates sampling at different times in the year (Edwards 1991), the survey period and intensity, although apparently inadequate for definitive inventory, served the purpose of comparing between trap designs, efficiency or capture of trappable fauna. Three parallel line transects, one dedicated for each extraction type (Berlese extraction method, pitfall trapping, and the Winkler methods) separated by 25 m inter-transect distances, were constructed north-southerly. The 25 m inter-transect distance between two consecutively set trap-transects was meant to minimize possible depletion effects, which can be caused by pitfall trapping (Digweed et al. 1995). Forty pitfall trapped samples and 40 litter samples each for Winkler and Berlese extraction were obtained. All samples were obtained on the same day between 08:00 and 09:30 h.

Each litter sample for Winkler and Berlese extraction was obtained by placing a 50 × 50 cm wooden frame on the forest floor and collecting the leaves, litter and loose humus from within the frame area into a large polythene bag (Frith and Frith 1990). Samples were obtained taking care to prevent possible escape of any invertebrate. The litter thus collected refers to the upper organic litter layer plus the loose humus layer. No underlying compact soil was obtained. Litter samples for Berlese and Winkler extractions were sieved in a 1.5 cm mesh wire sieve to separate larger materials of litter and transported to the laboratory in individual polythene bags.

Fauna was extracted with Berlese extraction apparatus (30.5 cm diameter, 35.6 cm height, 4–6 mm mesh screen, 25 w tungsten-filament lamp) over five days in 70% alcohol. Litter samples for Winkler extraction of the fauna (Besuchet et al. 1987) were placed in coarse-mesh bags, which were suspended inside a large sealed cloth bag suspended over a collecting bottle containing 75% ethanol. The litter and soil were left to dry at room temperature for five days. The litter material was gently mixed every day to ensure that the fauna remained active and to improve their chances of dropping into the collection cup (Parr and Chown 2001; Besuchet et al. 1987).

Each pitfall trap consisted of a black plastic bowl (210 mm diameter, 150 mm depth), buried to its rim in soil and partly filled with 30 mm of nontoxic, propylene glycol. Each trap was topped with a dark-plastic tray supported on iron bars to prevent either desiccation or flooding; such a system operated for 24 h continuously to avoid bias in catches arising from diurnal activity variation of fauna (Mommertz et al. 1996). Trapped fauna were separated, identified, counted, and the abundance and frequency of occurrence of taxa at each site was recorded.

The fauna obtained from the 40 litter samples from each method were available for data analysis. Taxa with > 30% frequency of occurrence in any of one of the sampling method was categorized as major, and the others as minor taxa. The sampling method, which trapped > 40% frequency of occurrence of a particular taxon, even if that taxon was trapped with < 40% frequency of occurrence in the other two methods was deemed to be ‘reasonably effective’ in sampling that particular taxon.

### Data analysis

Significant differences in the frequency of collection among sampling methods (abundance data with median and with low abundance and total absence of some taxa) made comparisons using common parametric statistics inappropriate. In the data analysis, emphasis was placed on seeking differences in the frequency of occurrence of arthropod types and less on testing for differences in the mean number of arthropod types (Prasifka 2007). Higher frequency of taxa obtained more frequently through a particular method than by the other two methods rendered that method more reliable. However, to summarize arthropod captures by trap type, the means and standard errors derived from individual trap were calculated for each arthropod group. To test for differences in the frequency with which particular arthropod taxa were collected by the three trap types, 2 × 3 contingency tables categorized each trap as either successful (one or more individuals collected) or unsuccessful (zero individuals collected); the differences were assessed with chi-square tests. Significant chi-squared values indicated an effect of trap type on the proportion of samples containing one or more individuals of an arthropod taxon (Prasifka et al. 2007). Trap-wise differences in the catch efficiency of individual taxa among the three trap types were assessed with a two sample z-test. Univariate comparisons through Kruskal-Wallis H tests were used to evaluate the significance level of trap-wise difference in faunal abundance. When significant differences were found, a Mann-Whitney U test was applied to determine which pairs of methods were different significantly (Weiss 2007). All the analyses were done using MegaStat Version 10.0 (Orris 2005).

**Figure 1.**
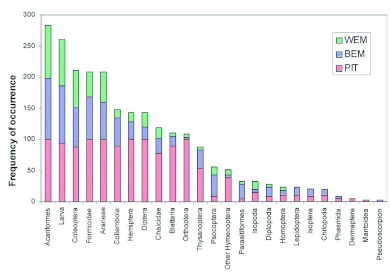
Percentage of abundance of ground dwelling arthropods collected from pitfall trapping (PIT), the Berlese extraction method (BEM), and the Winkler extraction method (WEM). High quality figures are available online.

## Results

**Figure 2.**
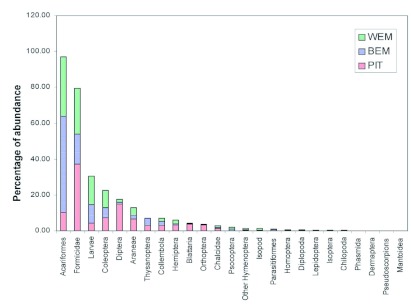
Percentage of frequency of ground dwelling arthropods collected from pitfall trapping (PIT), the Berlese extraction method (BEM), and the Winkler extraction method (WEM). High quality figures are available online.

From the three methods tested, 12,257 individuals belonging to 25 arthropod taxa were collected (Figure 1, Table 1), which could be broadly divided into (i) a major group of 14 arthropod taxa and one minor taxon, which showed significant differences in capture among the tested trapping methods and (ii) a minor group of 10 arthropod taxa with no difference in capture among the tested trapping methods (Tables 2 & 3). From among the 25 taxa, 18 occurred in all the three tested methods, whereas taxa belonging to Phasmida, Isoptera, Lepidoptera, Chilopoda occurred only in Berlese extraction and pitfall trapping methods, taxa belonging to Dermaptera and Mantoidea only occurred in pitfall trapping, and one taxon belonging to Pseudoscorpionida occurred only in Berlese extraction methods (Figure 1, Table 1). Based on the frequency of occurrence of fauna, the methods effectively trapped 13 major taxa in pitfall traps, nine major taxa and one minor taxa in the Berlese extraction method, and five major taxa in the Winkler extraction method (Figure 2, Table 1).

In the Winkler extraction method, 18 taxa were obtained. The proportionate distribution of dominant taxa in the collection was in the following order: Acariformes (85%) > insect larvae (75%) > Coleoptera (60%) > Araneae (48%) > Formicidae (40%) (Table 1). For the major taxa belonging to Coleoptera, Orthoptera, Blattaria, Hemiptera, Diptera, other Hymenoptera (except Formicidae and Chalcidae), Araneae, and Chalcidae, the Winkler extraction method separated the same frequency of occurrence as that of the Berlese extraction method, and for Psocoptera and Parasitiformes, the Winkler method obtained the same frequency of occurrence as that obtained in the pitfall trapping method (Table 2). Though the frequency of occurrence was lower than in other methods, 40% of occurrence (value set to consider any method as ‘reasonably effective’) of five taxa viz., Coleoptera, larval forms of diverse insect orders, Araneae, Acariformes, and Formicidae was obtained in the Winkler extraction method.

**Table 1.**
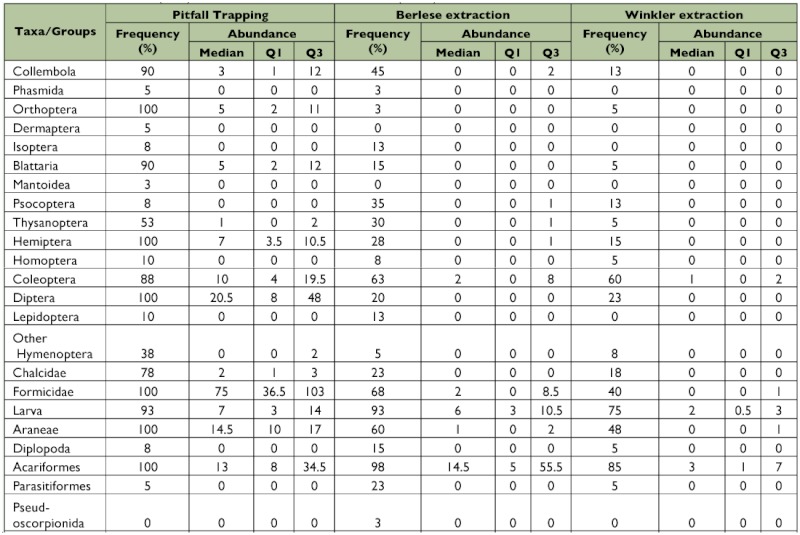
Frequency (%) and abundance (median + inter quartiles) of ground dwelling arthropods collected from pitfall trapping (PIT), the Berlese extraction method (BEM) and the Winkler extraction method (WEM).

In the Berlese extraction method, 23 taxa were obtained (Figure 2, Table 1). The dominance pattern of the major taxa was Acariformes (98%) > insect larvae (93%) > Formicidae (68%) > Coleoptera (63%) > Araneae (60%) > Collembola (45%) > Psocoptera (35%). The Berlese extraction method recorded the highest frequency of occurrence for major taxa belonging to Collembola, Thysanoptera, larval forms of diverse insect orders, Araneae, Formicidae, Psocoptera, and Acariformes and for one minor taxon, Parasitiformes, and an equivalent level of frequency of occurrence as Winkler extraction for 8 of the 14 taxa belonging to Orthoptera, Blattaria, Hemiptera, Coleoptera, Diptera, other Hymenoptera, Araneae, and Chalcidae (Table 2). A higher representation (> 40% of occurrence) of five taxa viz., Collembola, Coleoptera, larval forms of diverse insect orders, Araneae, and Formicidae was obtained in the Berlese extraction method.

In the pitfall trapping method, except Pseudoscorpionida, the remaining 24 taxa were recorded (Figure 2, Table 1).

Proportionate occurrence of the major taxa was in the order of: Acariformes = Formicidae = Orthoptera = Hemiptera = Diptera = Araneae > larval forms of diverse insect orders (93%) > Collembola (90%) > Blattaria (90%) > Coleoptera (88%) > Chalcidae (78%) > Thysanoptera (53%) (Figure 2, Table 1). For 11 out of the 14 taxa belonging to the Collembola, Orthoptera, Formicidae, Blattaria, Hemiptera, Coleoptera, Diptera, other Hymenoptera, Thysanoptera, Araneae, Chalcidae, Formicidae, the pitfall trapping method yielded the highest frequency of occurrence (Table 2). For two taxa (insect larvae and Acariformes) both pitfall trapping and Berlese extraction methods recorded same level of frequency of occurrence. Except for Psocoptera and Acariformes, the pitfall tapping method captured the highest frequency of occurrence for 13 out of the 14 major taxa (Table 2) and the same level of frequency of occurrence for 10 minor taxa.

**Table 2.**
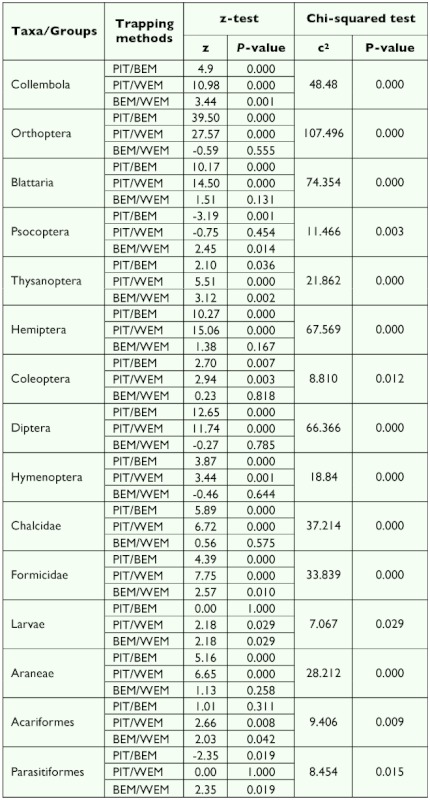
Results of Chi-squared test and two sample z- tests on the variation in the frequency of collection for ground dwelling arthropods using pitfall trapping (PIT), the Berlese extraction method (BEM) and the Winkler extraction method (WEM).

In summary, the pitfall trapping method yielded highest frequency of occurrence for 13 out of the 14 major taxa, Berlese extraction method for 3 out of the 14 taxa and also for one minor taxon, and the Winkler extraction method for none of the major taxa (Table 2). For the 10 minor taxa, all the three methods yielded the same level of frequency of occurrence. The arthropod abundance (median + inter quartiles) data for the ground-dwelling arthropod taxa in the three sampling methods is summarized in Table 1. Pitfall trapping recorded the highest abundance of 19 of the 25 arthropod taxa except for six taxa belonging to Psocoptera, Acariformes, Parasitiformes, Pseudoscorpionida, Chilopoda, and Diplopoda (Table 3). Abundance of all taxa was the lowest in Winkler extracted samples. High abundances of Blattaria, Diptera, and Orthoptera in the pitfall trapping and low abundances of these taxa in both Berlese and Winkler methods occurred.

## Discussion

Results of this study lead to recommendations on the relative usefulness of pitfall trapping, and Berlese and Winkler extraction methods for sampling ground-dwelling arthropods in subtropical, moist deciduous forests. A pronounced difference was found in the capture of major ground-dwelling arthropod taxa among the three tested sampling methods. Pitfall trapping yielded the maximal capture (both frequency and numbers) of 87% of the major taxa, followed by the Berlese extraction method (27%). The Winkler method proved ineffective for any of the major taxa in the moist, subtropical South Indian forest conditions. These percentages indicated that pitfall trapping was a useful standard arthropod collection method for ecological studies of ground-surface-dwelling arthropods compared with the Berlese and Winkler extraction methods. Irrespective of the method tested, few individuals of the minor taxa were captured and such an effect could be attributed to the low population densities of these taxa in the moist forests of the Western Ghats (Anu et al. 2009; Vineesh 2007; Anu 2006). Non-significant differences in the capture of minor taxa among the different trap types are difficult to interpret because of their low frequency of occurrence and abundance.

However, a bias was apparent in the samples obtained via pitfall trapping in comparison with the other two methods. Pitfall trapping captured high numbers of taxa active at the ground level, viz., taxa belonging to Orthoptera, Blattaria, Diptera, Araneae, Formicidae, Collembola, Hemiptera, Coleoptera, and other Hymenoptera (Prasifka et al. 2007; Leather and Watt 2005; Woodcock 2005; Bigknell et al. 2000), with 95–100%) frequency of capture when compared to their relatively low frequency of capture in the other two methods. On the contrary, with the Berlese sampling method, high capture was recorded of the less-active taxa associated with moisture and sheltered areas, viz., Acariformes, larvae of diverse insect orders, Psocoptera and Parasitiformes. Such variations and differences in the capture of taxa in relation to their surface activity is an established weakness of pitfall-trapping method, leading to the generalizations that (i) pitfall trap collections of arthropods are measures of activity rather than density estimates, (ii) pitfall trap extractions do not consider the resting and evasive behavior of many taxa thus leading to an under-representation of such taxa, and (iii) a comparison of data from pitfall traps with other methods is impossible in quantitative studies (Woodcock 2005; Oliver and Beattie 1996; Topping and Sunderland 1992; Adis 1979; Southwood 1978; Greenslade 1964). Moreover, unlike the other two methods, the pitfall trapping method captures arthropod taxa from an unknown area and calculations of absolute density of populations of taxa that is the number of individuals per unit of habitat is nearly impossible (Woodcock 2005; Work et al. 2002; Standen 2000; Spence and Niemela 1994; Halsall and Wratten 1988; Greenslade 1964). Calibrations and adjustments needed for the removal of these complex effects and standardization of data would diminish the time and labour efficiency advantages of using pitfall trapping (Stoyan and Kuschka 2001; Mommertz et al. 1996; Spence and Niemela 1994; Topping and Sunderland 1992). The results support the earlier findings and since the data from pitfall trapping are not useful for estimating absolute abundance (populations per unit area or volume) in multigroup ecological approach involving ground surface dwelling arthropods, it would be prudent to limit their use for production of qualitative data. These setbacks (viz., sampling biases and interpretational difficulties) make density-based estimates from quadrat sampling methods (Berlese or Winkler extraction), which measure populations in numbers of animals/unit area as a better alternative for quantitative multitaxa ecological studies of ground-dwelling arthropods.

**Table 3.**
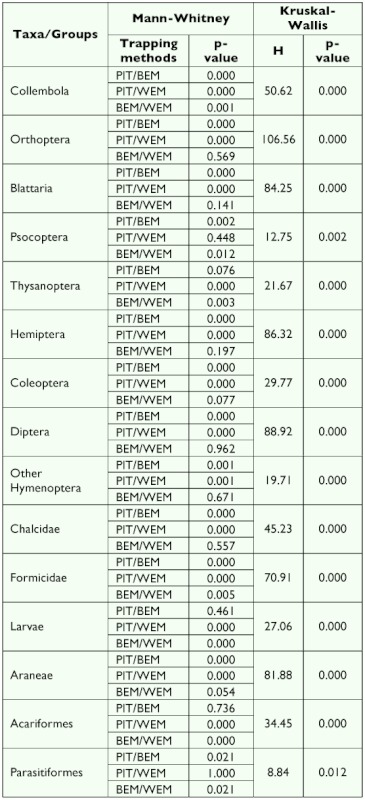
Results of Kruskal Wallis and Mann-Whitney tests on the variation in the abundance of collection for ground dwelling arthropods using pitfall trapping (PIT), the Berlese extraction method (BEM) and the Winkler extraction method (WEM).

Unlike the Berlese or Winkler litter extraction methods, pitfall trapping requires two trips to interior forests: one to set the traps and the second to retrieve traps and samples. A second field trip to retrieve the traps leads to additional expenditure, loss of time and practical difficulties in protected forests with restricted access. Moreover, multiple chances of wildlife disturbing the field-placed traps and inclement weather affecting the sampling effort in subtropical forests also exist. This leaves the researcher apprehensive about the success of his collection efforts until the second trip and makes pitfall trapping more laborious and chance oriented than the other two methods.

Recent studies have highlighted the Winkler extraction method as a less expensive, more convenient, and a more efficient alternative method for exhaustive extraction of soil macro-invertebrates (Krell et al. 2005; Didham et al. 1998; Nadkarni and Longino 1990). Hence, at the beginning of this study, it was expected that the Winkler extraction method would prove better than the pitfall trapping and Berlese extraction methods by obtaining greater numbers and frequency of different arthropod taxa. However, an entirely different outcome became evident. Firstly, the Winkler extraction method underestimated the abundance and frequency of the major taxa (in South Indian moist deciduous forest conditions), except taxa belonging to Coleoptera, larvae of multiple insect orders, Araneae, Acariformes, and Formicidae; secondly, most of the minor taxa belonging to Dermaptera, Mantoidea, Pseudoscorpionida, Phasmida, Isoptera, Lepidoptera, and Chilopoda were missed out completely. These limitations highlight the less emphatic observations of Besuchet et al. (1987) that Winkler extraction method is less suitable for the extraction of all taxa, and there is possibility of death of taxa with a narrow ecological tolerance before dropping into the collection jars. Although Winkler extractions are cost effective and convenient with limited time needed to sort fauna (Krell et al. 2005) and effective in studies of litter Formicidae and Coleoptera (Fisher 2004; Fisher and Robertson 2002; Didham et al. 1998; Olson 1991; Nadkarni and Longino 1990; Besuchet et al. 1987), the limited volume of quantitative information they generate for the majority of ground surface dwelling arthropod taxa weaken their value as an effective individual sampling method for intensive ecological studies of ground-dwelling arthropod fauna. Despite that, the Berlese extraction method will be costly and laborious because it requires more time to sort out the fauna from fallen debris and soil in the laboratory (Snyder et al. 2006). Berlese extraction recorded higher capture of taxa per unit volume than the Winkler extraction method. Greater efficiency and thoroughness of the sampling effort makes the Berlese extraction method a better choice than the Winkler extraction method as an individual quantitative sampling method for the ground surface dwelling fauna in a subtropical moist deciduous forest.

Group and trap specific differences noted in the present study supports the findings by Edwards (1991) and Standen (2000) that no single extraction method is the best for all taxa of ground-dwelling arthropods, and it may be necessary to use more than one method based on the aim of the study. The selection of sampling methods for ground-dwelling arthropods should be made based on the data — quantitative or qualitative — required for the study. High trapping efficiency of a majority of the taxa makes pitfall trapping the best method in qualitative inventory studies of ground-dwelling arthropods, but not for quantitative studies because of the above-cited setbacks. Trapping success of pitfall traps conforms to the findings (Spence and Niemelä 1994) that pitfall trapping remains the only realistic way to survey large acreages where qualitative inventory and a comparison of species assemblages of ground-active arthropods is required. However, for quantitative studies of ground-dwelling arthropods, the Berlese extraction method is the best option. Very high abundance and frequency of occurrence of Blattaria, Diptera and Orthoptera in pitfall trapping and very low trapping with the Berlese extraction method suggest that a combination of pitfall trapping and Berlese extraction and standardization of pitfall trapping data (Stoyan and Kuschka 2001) is more feasible for exhaustive quantitative studies of surface dwelling arthropods.

## Conclusions

The relative abundance and frequency of occurrence of fauna was different with the three sampling methods. When cost and time constraints dictate the limiting of ground-dwelling arthropod sampling to one method, the Berlese extraction method is ideal for quantitative estimates, and the pitfall trapping method is ideal for qualitative estimates.

Since the three taxa (Orthoptera, Diptera and Blattaria) with a low catch probability with Berlese extraction were caught efficiently with pitfall trapping, inclusion of pitfall trapping with appropriate adjustments would be the method for comprehensive quantitative surveys of ground-dwelling arthropods. Although pitfall trapping samples tend to include more ground-active species, its efficiency indicates that pitfall trapping is certainly the method of choice for an individual quantitative sampling method for most major taxa except the Psocoptera and insect larval forms, for which the Berlese extraction method is a better option.

As a cost effective, individual quantitative sampling method, Winkler extraction is suitable for obtaining Coleoptera and Acariformes in addition to litter Formicidae, for which it is an established method (Delabie et al. 2007; Underwood and Fisher 2006; Longino et al. 2002; Bestelmeyer et al. 2000; Delabie et al. 2000), but not for ecological studies involving multiple arthropod groups.
